# Evaluation of fetal foramen ovale blood flow by pulsed Doppler ultrasonography combined with spatiotemporal image correlation: To define the normal reference range of fetal foramen ovale blood volume for each gestational age: a cross-sectional study

**DOI:** 10.1186/s12947-021-00247-0

**Published:** 2021-05-05

**Authors:** Wenjuan Tang, Yuanchen Luo, Shi Zeng, Jiawei Zhou, Ganqiong Xu, Jianwen Yang

**Affiliations:** 1 Department of Ultrasound Diagnosis, The Second Xiangya Hospital of Central South University, 139 Renmin Road (M), Changsha, 410,011 China; 2 Department of Ultrasound Diagnosis, The First Hospital of Changsha, Changsha, China; 3 Department of Ultrasound Diagnosis, The Third People Hospital of Yongzhou, Yongzhou, China

**Keywords:** Foramen ovale, Blood flow, Spatiotemporal image correlation, Doppler

## Abstract

**Objective:**

The objective of this study was to determine fetal foramen ovale blood flow utilizing pulsed Doppler combined with spatiotemporal image correlation.

**Methods:**

A cross-sectional study was performed in 440 normal fetuses between 20 and 40 weeks of gestation. In order to calculate foramen ovale blood flow, the foramen ovale flow velocity–time integral was obtained by pulsed Doppler ultrasonography, and the foramen ovale area was measured by using spatiotemporal image correlation rendering mode. Foramen ovale blood flow was calculated as the product of the foramen ovale area and the velocity–time integral.

**Results:**

Gestational age-specific reference ranges are given for the absolute blood flow (ml/min) of foramen ovale, showing an exponential increase from 20 to 30 weeks of gestation, and a flat growth trend during the last trimester, while the weight-indexed flow (ml/min/kg) of foramen ovale decreased significantly. The median weight-indexed foramen ovale blood flow was 320.82 ml/min/kg (mean 319.1 ml/min/kg; SD 106.33 ml/min/kg).

**Conclusions:**

The reference range for fetal foramen ovale blood flow was determined from 20 to 40 weeks of gestation. The present data show that the volume of foramen ovale blood flow might have a limited capacity to increase during the last trimester.

## Introduction

Shunting through the foramen ovale (FO), which constitutes the majority of left ventricular cardiac output (LVCO), is critical for the delivery of enriched oxygenated blood to the coronary circuit, cerebral circuit, and upper body of the fetus [1]. Premature closure or restriction of the FO leads to reduced or restricted right-to-left atrial shunting. There is substantial evidence that primary closure of the FO results in hypoplasia of left heart structures [2, 3]. Studies of animal [4, 5] and human fetuses [6–9] have also demonstrated that adaptive changes in FO blood flow volume occur during hypoxia or hypovolemia, suggesting that quantifying FO blood flow and its redistribution plays an important role in assessing fetal adaptation to nutrient and oxygen deficiency. The combination of high-resolution imaging and Doppler ultrasonography has provided a method for calculating the volume of blood flow in the heart and great vessels as the product of the Doppler flow velocity–time integral (VTI) and the cross-sectional area of the flow stream [10]. Because the FO has an irregular shape and a multiphasic blood velocity waveform during the cardiac cycle [11], FO blood flow volume was estimated previously by subtracting pulmonary blood flow (QP) from LVCO [1, 12–14]. However, indirect assessment of FO blood flow volume resulted in varied data due to different measurement methods for QP used by different authors. In addition, errors arising from inaccuracies in vessel diameter measurement and Doppler recording are inevitable, especially in small-diameter vessels such as the ductus arteriosus or the left and right pulmonary arteries.

Four-dimensional ultrasound (4DUS) involves the capture of multiple adjacent two-dimensional planes to recreate a four-dimensional volume. Spatiotemporal image correlation (STIC) software captures a fetal heart volume with a single automated sweep of the transducer in a limited time. Spatial and temporal information are combined to display dynamic images that can be extracted from volume datasets. STIC allows heart anatomy visualization through a cine loop sequence in multiplanar and rendering modes. Furthermore, fetal heart rate (FHR) can be detected and synchronized two-dimensional images. STIC can produce more standardized imaging of the fetal heart and reduce operator dependency compared with conventional two-dimensional ultrasound (2DUS) [15]. Visualization of specific heart structures by surface rendering may provide an additional method for assessing cardiac morphology and function and has been used to calculate the area of many fetal cardiac structures, and these measurements present good intra- and inter-observer reproducibility [16–19].

Few published data exist on normal FO blood flow volume in relation to fetal gestational age. Thus, the present study was designed to calculate FO blood flow volume directly as the product of foramen ovale area (FO-A) and foramen ovale flow velocity-time integral (FO-VTI). The FO-A was measured by using 4DUS with STIC in rendering mode, and the FO-VTI was obtained by pulsed Doppler. The objective of this study was to define the normal reference range of FO blood flow volume for each gestational age.

## Materials and Methods

### Study Population.

Healthy women with a singleton fetus at 20–40 weeks of gestation were recruited from the Second Xiangya Hospital, Central South University, and the Third People Hospital of Yongzhou, Hunan, between April 2019 and August 2020 for a cross-sectional study. The inclusion criteria of the study population were as follows: the fetus had no cardiac or extracardiac malformation; each fetus was appropriate for its gestational age in size (between 10 and 90th percentile growth curves for local standards); the amniotic fluid volume was normal; and the mother was in good health and free of diabetes, hypertension, proteinuria, smoking, and drug use. The Research Review Committee of our institution approved the research protocol, and each participant gave written consent to join the study.

### Measurements.

The participants were examined using a Voluson E8 device (General Electric, Healthcare, Zipf, Austria) equipped with a 2.0 or 5.0 MHz RAB 4-8L convex probe. All measurements were performed in the state of fetal quietness without fetal respiratory movement. All the recordings were performed by an examiner (W.J.T) with expertise in the 4DUS for obstetrics who received 1 year of specific training in the evaluating and measuring FO-A.

**Fig. 1:**
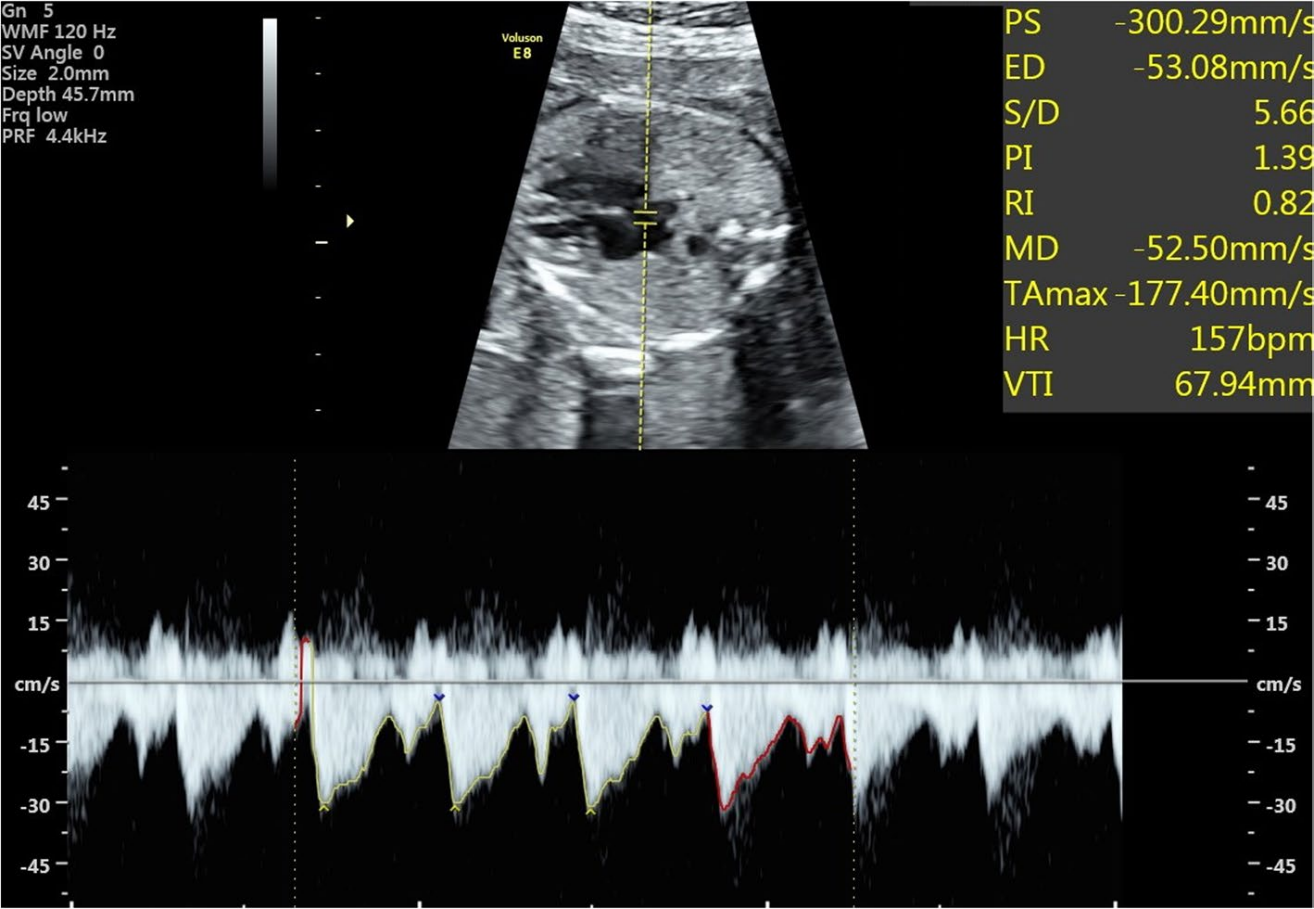
Measurement of the foramen ovale blood velocity waveforms. The Doppler sample volume is located within the left atrium and is parallel to the transarterial flow. The transforaminal flow is bidirectional, with right-to-left flow predominating

Initially, a 2DUS examination was performed to evaluate fetal biometry, including the biparietal diameter, head circumference, abdominal circumference, and femoral length, and to calculate the estimated fetal weight (EFW, in kilograms). Gestational age was determined by the date of the last menstrual period and confirmed by sonographic biometry in early pregnancy; Image-directed pulsed and colour Doppler equipment was used to obtain the blood velocity waveforms at the level of the FO, and the sample volume was placed on the left atrial side of the four-chamber view (Fig. 1). A 120 Hz high-pass filter was used, and the spatial-peak temporal average power output for colour and pulsed Doppler was kept at < 100 mW/cm^2^. The sample volume was set at 2-3 mm. If there was an angle of < 15° between the direction of FO blood flow and the Doppler beam, the data were included in the analysis. From Doppler traces, the FHR (in beats per minute) was measured, and the FO-VTI (in millimeters) was determined by measuring the area underneath the Doppler spectrum with a planimeter. Only the right-to-left flow measurements were reported. At least three consecutive cardiac cycles were analysed, and their mean value was used for further analysis. Next, the right and left atrial transverse diameter at end of systole (in millimeters) and the length of atrial septum (in millimeters) were measured in the same four-chamber view.

**Fig. 2:**
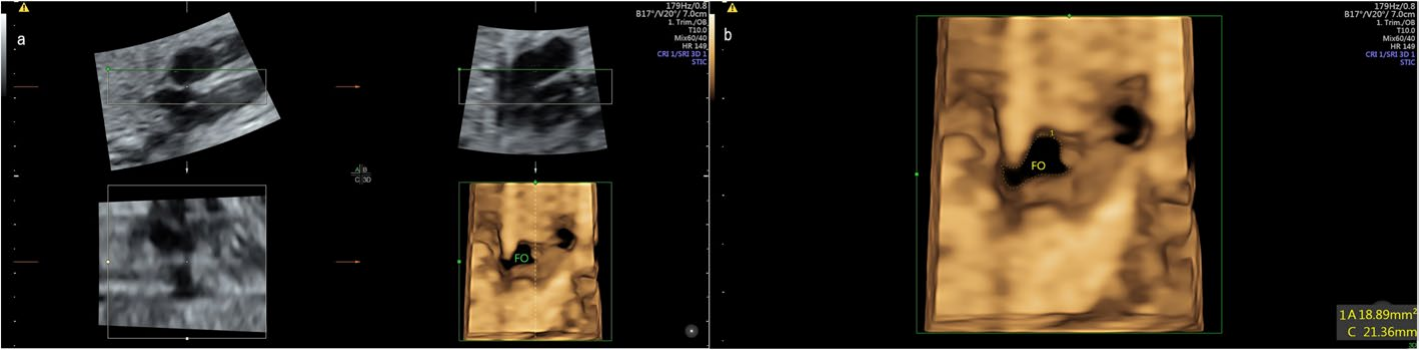
**a** Measurement of the foramen ovale area using the cardio-STIC application in rendering mode. A four-chamber plane shown in a multiplanar image with the region of interest (green line) positioned on the atrial septum of the heart (plane A), rendering an image of the foramen ovale at its maximum opening (3D plane). **b** Magnified rendering image showing the foramen ovale, with the measurement of its area

Subsequently, the fetal FO-A (in millimeters squared) was calculated using the cardio-STIC application. The four-chamber view was used as a reference. The optimal position of the fetal spine for imaging was approximately 3 or 9 o'clock. The sampling frame was adjusted to encompass the entire fetal heart with all its vascular connections at the smallest possible dimension. An aperture angle of 20–40°was used according to gestational age, and the acquisition time was 10–12.5 s. Each mother was asked to hold her breath during acquisition time until a single four-dimensional volume was collected. Later, offline evaluation of the STIC volumes was performed by one of the authors (W.J.T). The four-chamber view was selected as a reference plane, and it was rotated around the Z-axis until the atrial septum and ventricular septum were both in a horizontal position. The rendering button was pressed, and the device was set to surface mode. The reference dot was positioned at the level of the FO and the region of interest (ROI) was adjusted to encompass the entire atrial septum and ventricular septum with the green line of ROI positioned inside the right atrioventricular cavity. The size of the ROI was modified to include half of the left and right atriums. Each section was shown layer by layer from the right atrial side until the FO was clearly visible (Fig. 2a). FO-A was measured at its maximum opening. Next, the rendered image was enlarged and shown on the full screen, the measure button was activated, and the area was manually painted along the margin of FO  (Fig. 2b).

### Blood flow calculations.

The absolute blood flow of foramen ovale (QFO, in milliliters per minute) was calculated with the following formula: QFO = FHR × FO-A × FO-VTI.

Next, QFO was normalized to EFW. Weight-indexed foramen ovale blood volume (iQFO, in milliliters per minute per kilogram) was calculated by using the following formula: iQFO = QFO/EFW.

For the assessment of inter-observer reliability, a second examiner (J.W.Y) with the same level of experience in 4DUS in obstetrics performed a second measurement on QFO of 80 randomly chosen fetuses. The examiners were not allowed to share results.

### Statistical analysis

For descriptive analysis, the means, medians, standard deviations, and maximum and minimum values of the FO-A and FO blood volume for each gestational age were determined. A curvilinear regression model was fitted to the data versus gestation age, and to interpolate FO-A versus EFW. Bland–Altman plots were used to assess inter-observer variability. Statistical analysis was performed using SPSS 25.0. For all the analyses, statistical significance was defined as P < 0.05.

## Results

A total of 550 participants underwent fetal echocardiography, of whom 110 were removed from the data set due to the poor technical quality of their fetal cardiac images, which was caused by maternal conditions including obesity and abdominal scars. The concentration of losers in the third trimester led to a 69% (253/363) of the viewing rate for FO-A in late pregnancy. Adequate datasets for subsequent analysis were ultimately obtained in 440 fetuses (80% rate of acceptance). Each fetus was delivered at term and had an uncomplicated neonatal course. The mean gestational age (with SD) was 29.6 ± 5.7 weeks, the mean maternal age was 27.9 ± 6.0 years (range 17–42), the mean maternal weight was 55.49 ± 4.18 kg, and the mean maternal height was 159.97 ± 4.37 cm.

**Fig. 3:**
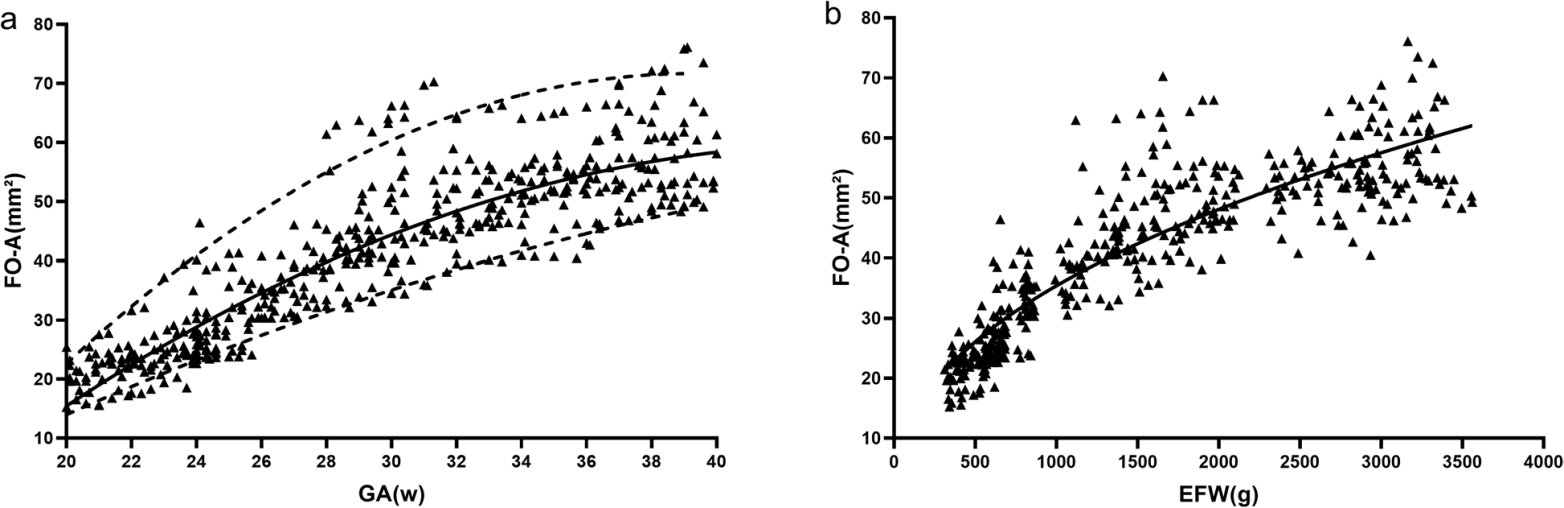
**a** A significant correlation exists between gestational age (GA) and fetal foramen ovale area (FO-A). The FO-A is presented as the 5th, 50th, and 95th percentiles. The equation for the 50th percentile was FO-A = -87.26 + 6.63GA-0.07GA^2^; r = .91, P < .0001. **b** Changes of fetal foramen ovale area (FO-A) vs. estimated fetal weight (EFW). (Regression slope of FO-A = 1.20EFW^0.49^, r = 0.84, P < .0001)

The mean FO-A was 20.53 ± 2.79mm^2^ (range 15.25–25.47) in the 20th week of pregnancy and 57.85 ± 7.81mm^2^ (range 49.12- 76.14) in the 40th week of pregnancy. In STIC rendering mode, the FO presented a round, oval, or irregular shape. The growth of the FO-A increased significantly before 30 weeks, followed by slower growth (Fig. 3a). Figure 3b showed increasing area of the FO versus EFW. The power exponent of the FO-A curve indicated that the increase in FO-A was approximately the square root (0.49) of the fetal body weight.

**Fig. 4:**
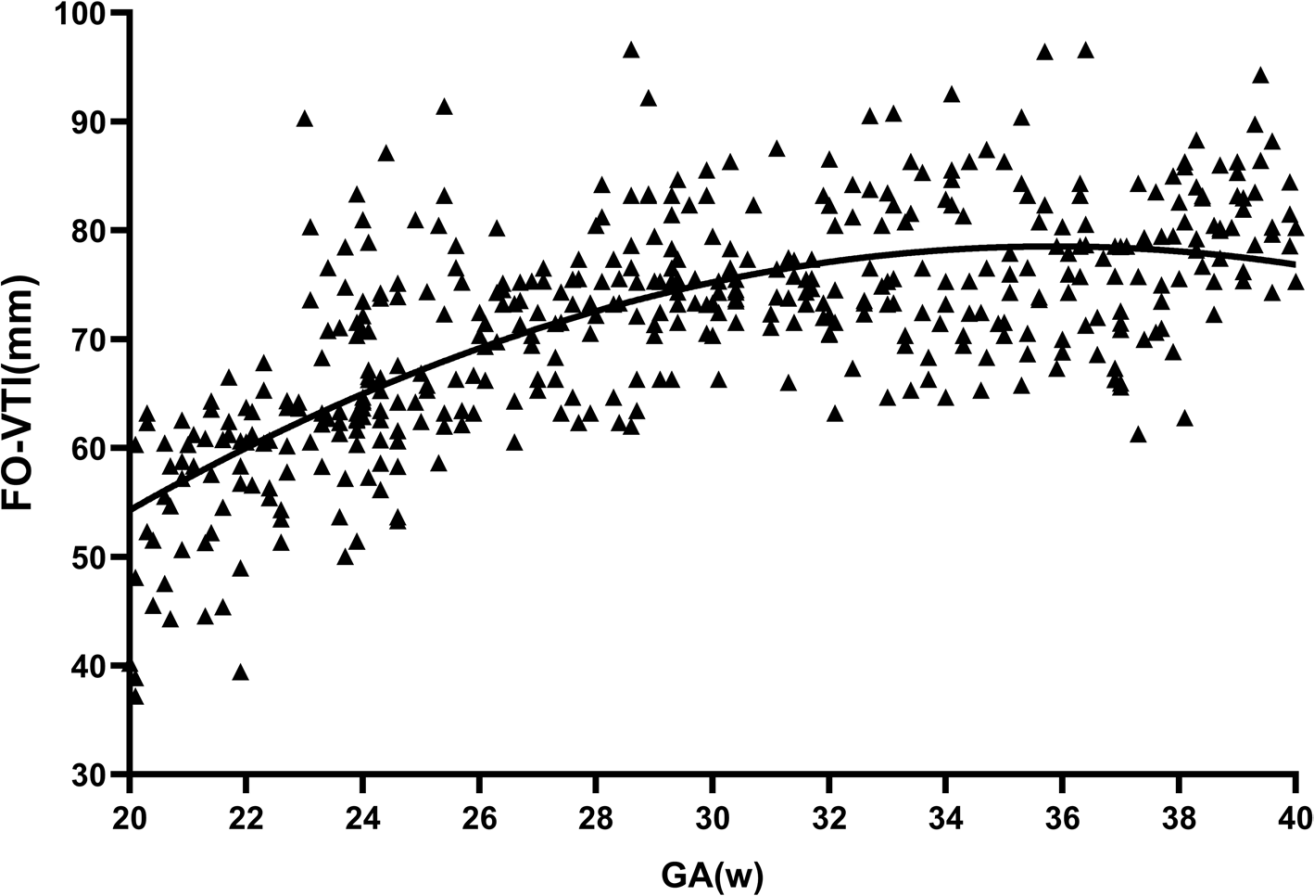
Regression slopes show a good correlation between gestational age (GA) and foramen ovale flow velocity–time integral (FO-VTI). (Regression slope of FO-VTI = -45.55 + 6.92GA -0.09GA^2^; r = .70, P < .0001)

The median FHR derived from Doppler recording in the FO was 141 bpm (mean 142 bpm; SD 9.11 bpm). Figure 4 demonstrated the trend in FO VTI with advancing gestational age.

General echocardiographic findings, 5th, 50th, and 95th percentiles for the QFO and iQFO at each gestational age evaluated were shown in Table 1.

**Table 1:** General echocardiographic finds and the 5th, 50th, and 95th percentiles of fetal foramen ovale absolute blood flow (ml/min) and weight-indexed foramen ovale blood volume (ml/min/kg) between the 20th and 40th weeks

					QFO (ml/min)				iQFO (ml/min/kg)			
GA	n	RALD	LALD	ASL	SD	5th	50th	95th	SD	5th	50th	95th
20–20^+6^	20	5.61 ± 0.29	5.38 ± 0.31	9.94 ± 0.91	36.30	93.31	160.10	212.37	108.32	221.14	453.56	636.34
21–21^+6^	21	6.11 ± 0.58	5.83 ± 0.58	10.29 ± 0.98	34.84	123.47	191.39	230.49	89.66	252.55	445.09	568.94
22-22^+6^	21	6.54 ± 0.42	6.19 ± 0.34	10.58 ± 0.69	27.19	155.12	208.82	254.24	57.13	292.09	401.58	470.93
23–23^+6^	29	7.21 ± 0.59	6.79 ± 0.49	10.85 ± 0.64	37.53	157.62	242.61	300.78	76.82	271.65	396.41	542.66
24–24^+6^	36	7.48 ± 0.64	7.08 ± 0.65	11.09 ± 0.64	54.35	211.55	260.12	419.32	79.22	321.14	402.91	609.29
25–25^+6^	20	7.78 ± 0.62	7.28 ± 0.58	11.19 ± 0.67	28.55	264.13	313.88	365.91	54.09	333.91	412.44	516.23
26–26^+6^	20	8.15 ± 0.65	7.68 ± 0.78	11.29 ± 0.56	25.86	304.34	352.35	399.10	35.24	352.79	417.53	476.38
27–27^+6^	20	8.75 ± 0.65	8.08 ± 0.65	11.39 ± 0.52	32.68	308.05	371.94	450.99	35.74	279.05	341.14	410.61
28–28^+6^	23	9.43 ± 0.69	8.68 ± 0.76	11.48 ± 0.56	78.43	305.97	419.07	580.45	74.10	232.39	344.69	519.34
29–29^+6^	26	9.97 ± 0.81	9.42 ± 0.69	11.58 ± 0.55	60.23	381.06	489.94	591.04	39.67	250.94	342.00	411.94
30–30^+6^	20	10.42 ± 0.65	9.68 ± 0.62	11.64 ± 0.65	69.93	389.49	491.07	606.57	45.93	244.19	320.79	399.09
31–31^+6^	20	10.60 ± 0.71	9.78 ± 0.66	11.76 ± 0.61	54.34	405.23	498.51	607.57	45.25	213.89	306.44	390.30
32–32^+6^	20	10.94 ± 1.09	10.09 ± 0.96	11.83 ± 0.44	56.31	410.44	514.63	612.74	37.99	215.73	270.29	339.48
33–33^+6^	20	11.61 ± 0.86	10.83 ± 1.00	11.91 ± 0.38	42.26	450.88	517.82	613.29	23.52	219.17	262.57	312.62
34–34^+6^	20	12.07 ± 0.83	11.33 ± 0.67	12.13 ± 0.39	43.47	458.19	524.65	628.23	20.97	178.39	212.26	261.95
35–35^+6^	20	12.50 ± 0.82	11.89 ± 0.86	12.17 ± 0.41	57.58	423.19	531.33	636.65	29.49	141.25	205.03	250.59
36–36^+6^	21	12.83 ± 0.60	12.11 ± 0.46	12.22 ± 0.39	72.63	451.00	560.04	684.66	29.73	147.63	197.39	259.44
37–37^+6^	21	13.37 ± 0.57	12.61 ± 0.79	12.27 ± 0.32	66.73	452.52	565.39	724.57	22.39	155.80	182.68	229.17
38–38^+6^	21	13.96 ± 0.64	13.12 ± 0.71	12.29 ± 0.43	76.68	472.33	578.14	743.19	24.71	154.04	184.86	233.39
39–40	21	14.46 ± 0.63	13.66 ± 0.65	12.41 ± 0.54	78.09	491.03	576.07	752.35	25.71	140.99	179.97	237.64

Data expressed as mean ± standard deviation

*GA* gestational age, *n* number of pregnant women at each gestational age, *SD* standard deviation, *QFO* Foramen ovale absolute blood flow, *iQFO* Weight-indexed foramen ovale flow, *RALD* Right atrial transverse diameter at end of systole, *LALD* Left atrial transverse diameter at end of systole, *ASL* Length of atrial septum

**Fig. 5:**
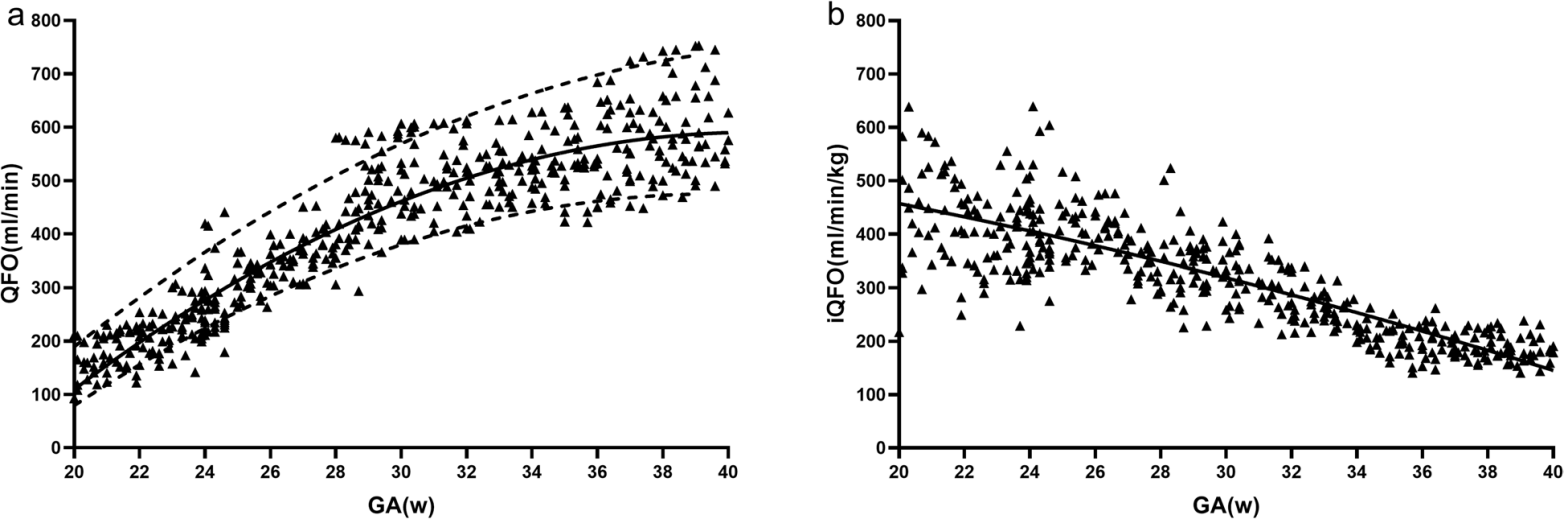
**a** Foramen ovale absolute blood flow (QFO) is presented as the 5th, 50th, and 95th percentiles. The equation for the 50th percentile was QFO = -1262.39 + 90.87GA-1.11GA^2^; r = .93, P < .0001. GA, gestational age. **b** Regression slopes show a significant correlation between gestational age (GA) and weight-indexed foramen ovale blood volume (iQFO). (Regression slope of iQFO = 629.20 -5.11GA -0.17GA^2^; r = .84, P < .0001)

Fetal QFO increased more than threefold from 20 to 40 weeks of gestation. Figure 5a showed the QFO increased significantly from 20 to 30 weeks of gestation (the mean growth rate was 67%) and then showed a slow upward trend between 30 and 40 weeks of gestation (the mean growth rate was 17%). Figure 5b presented the trend of iQFO with gestational weeks. The negative slope of iQFO was significantly from 20 to 40 weeks of gestation, mean iQFO decreased by 59% (from 450.30 ml/min/kg at 20 weeks of gestation dropped to 184.91 ml/min/kg at term). Median iQFO was 320.82 ml/min/kg (mean 319.1 ml/min/kg; SD 106.33 ml/min/kg).

**Fig. 6:**
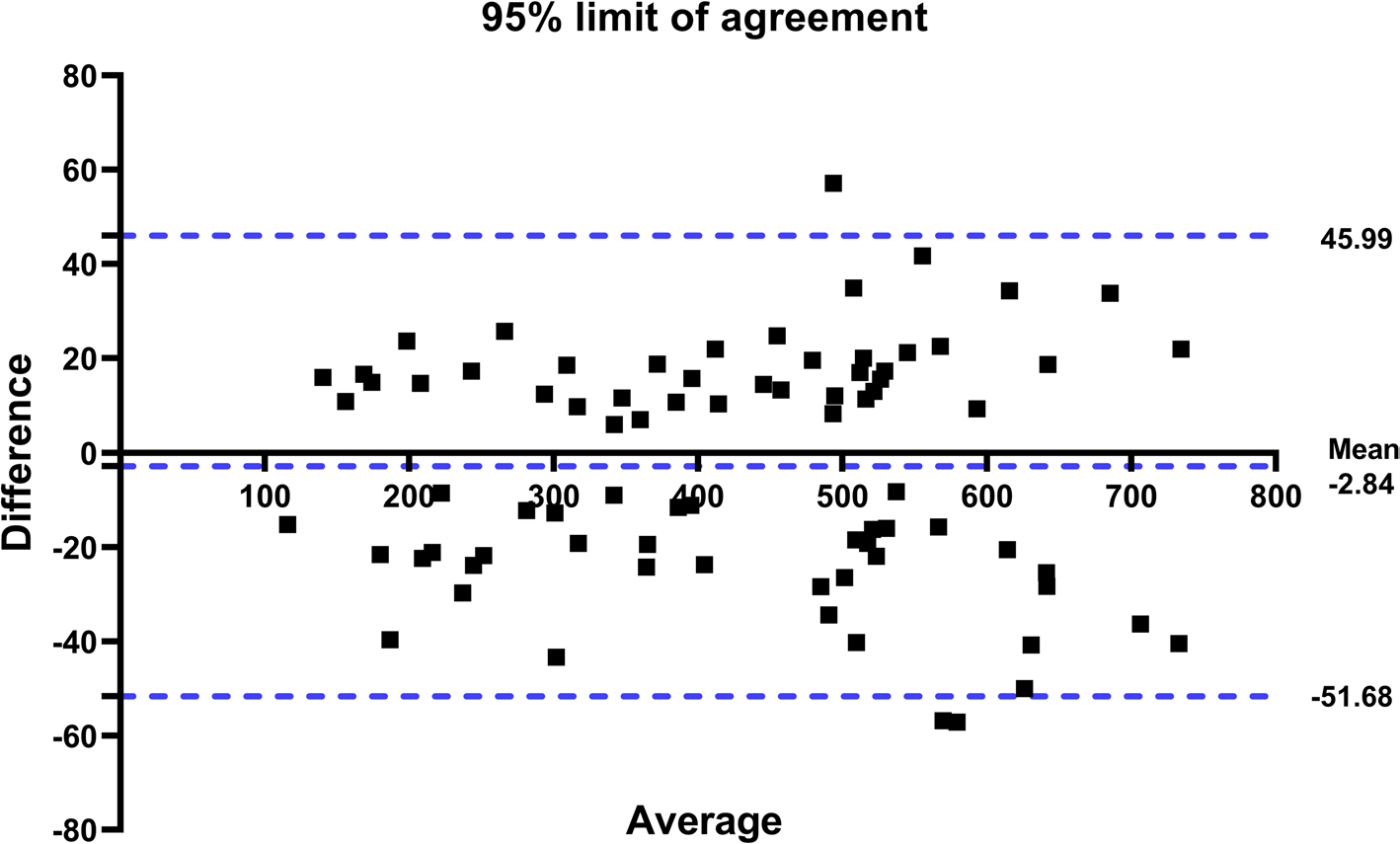
Bland–Altman plots showing the mean difference between two measurements of fetal foramen ovale absolute blood flow made by two examiners (inter-observer) plotted against the difference in their means

Through the Bland–Altman plots, good agreement in the measurement for the QFO was observed, with a mean difference of 2.84 (SD ± 24.92 ml/min and 95% CI ± 48.84 ml/min) for inter-observer reliability (Fig. 6).

## Discussion

Our study identified gestational age-specific reference ranges for FO blood volume in normal fetuses from 20 to 40 weeks of gestation using STIC in rendering mode combined with pulsed Doppler. The findings of this study suggest that QFO increases with advancing gestational age, showing an exponential increase from 20 to 30 weeks of gestation and a flat growth trend during the last trimester, while iQFO decreases with advanced gestational weeks.

The present study showed that QFO increased more than threefold from 20 to 40 weeks of gestation. QFO showed an exponential increase between 20 and 30 weeks of gestation, the mean growth rate was 67%, whereas the mean growth rate was only 17% from 30 to 40 weeks of gestation. In fetal circulation, enriched oxygenated blood originating from the placenta enters the fetus via the umbilical vein, and part of this constitutes the majority of LVCO through the FO. Therefore, the growth of QFO was associated with an increase in LVCO with advancing gestational age. The emergence of a period of slow growth may be related to the increase in QP in the third trimester. In a study investigating the relationship between QFO and QP in the proportion of LVCO composition in human fetuses during pregnancy, Rasanen et al. [14]found that QP constituted 27% of the LVCO at the beginning of the second half of pregnancy and 50% of the LVCO during the third trimester. The filling of the left atrium is enhanced by an increase in pulmonary venous return, such that right-to-left shunting through the FO may be limited due to the elevation of left atrial pressure. Animal studies have also demonstrated that QFO cannot fully compensate for impaired pulmonary venous return [20, 21], and FO has limited ability to increase its volume blood flow at near-term gestation [22].

There was a declining trend in iQFO with advancing gestational age in our study. Mean iQFO decreased by 59%. The decrease of iQFO with advanced gestational weeks may be related to the decrease of ductus venosus shunt in addition to the increase of QP. Bellotti et al. [23] found the weight-indexed amniotic umbilical flow did not change significantly during gestation, whereas weight-indexed ductus venosus flow decreased significantly, the percentage of umbilical blood flow shunted through the ductus venosus decreased significantly (from 40 to 15%), consequently, the percentage of flow to the liver increased between 20 and 38 weeks gestation. Rudolph et al. [24] found that 55% of the umbilical blood flow was shunted through the ductus venosus in 33 exteriorized human fetuses at 10 to 20 weeks gestation by using isotope-labelled microspheres, and Kiserud et al. [25] found only 20% to 30% shunting through the ductus venosus for the direct supply of the FO during the second half of pregnancy by ultrasonic measurement; correspondingly, an average of 70% to 80% of the oxygenated umbilical venous blood perfused the hepatic tissue. As fetal growth accelerates, an increased proportion of umbilical blood is directed to the liver, and the proportion that is shunted through the ductus venosus decreases, although the blood flow in the umbilical vein grows as pregnancy progresses. The reduction of shunting through the ductus venosus to the inferior vena cava limits, to some extent, the capacity of the FO to increase its volume of blood flow in the third trimester. At the same time, the increasing diversion from cardiac to pulmonary circulation enhances the output of left ventricle as gestation advances, although the reflux of deoxygenated blood from the lungs to the left atrium through the pulmonary veins is marginal in early pregnancy. Phase contrast magnetic resonance imaging (PC-MRI) is a promising new technique for the study of the fetal circulation. It has been shown to be feasible in measurement of the vessel blood flow in the fetal lamb [26] and late-gestation human fetus [27–30], and has been used successfully to make initial observations of redistribution of the fetal circulation in human fetuses with congenital heart disease [31, 32]. The mean values of iQFO in the third trimester calculated in the present study are slightly higher than previous PC-MRI measurements in a small series of human fetuses in late pregnancy [27, 28]. This may be because ultrasound technique assumed a constant flow velocity across the vessel lumen, where in fact the flow was likely slower around the vessel periphery than the flow velocity sampled in the middle of the vessel. The Doppler flow parameter used to quantify blood flow in our study is VTI which is the distance that the maximum flow velocity of the stream travels per unit time regardless of the distribution gradient of blood flow velocity in the vessel, this parameter may lead to the overestimation of iQFO to some extent. In addition, intrinsic inaccuracies in ultrasound measurements of flow may also account for discrepancy, such as the measurement of the cross-sectional area and ultrasonic beam angle correction [33]. The mean iQFO (319.1 ± 106.33 ml/min/kg) in the present study is higher than fetal lamb study [26] in which the mean iQFO was 164 ± 20 ml/min/kg. The possible reason is the different proportion of brain mass in animal and human fetuses in addition to the above factors.

Analysing the growth pattern of FO is helpful to understand the variation trend of QFO. In the present study, the FO-A was based on direct measurements in STIC rendering mode. The data showed linear increases with advancing gestation, but slow growth was seen after 30 weeks of gestation. This growth pattern is determined by changes in blood flow through the FO during different gestations, the FO-A increases proportionally to the absolute blood flow to the FO. The data for the FO-A were slightly higher than those in a previously published report [34], in which the horizontal area between the FO valve and atrial septum, as calculated by 2DUS, grew from 15 to 50 mm^2^ between 18 and 42 weeks of gestation. However, Patten et al. found that the area of the orifice in the atrial septum was larger than the horizontal area between the FO valve and atrial septum in fetuses and new-borns [35], which corresponds closely to our results. The difference in measurement methods may be a factor contributing to the discrepancy. The sagittal view of the FO in the atrial septum not routinely feasible with 2DUS can be generated by STIC. It is possible to directly observe and measure the size of FO by using STIC. Yagel et al. [36] have also suggested that the rendering mode enables superior evaluation of FO in the interatrial septum. Furthermore, STIC offers the possibility of offline analysis without the need for the pregnant woman to be present [37, 38]. In our study, a section including all four heart chambers was easily obtained as the initial scanning surface. As long as the professional has been appropriately trained to sample cardiac volumes, these volumes can be obtained without the need for a specialist, which does not become essential until later, to identify possible abnormalities of heart morphology [38]. An interesting observation was made when we compared FO-A with EFW, FO-A increases approximately as the square root (0.49) of the EFW. Our finding clearly demonstrates that the increase in FO-A is proportional to body size.

The Doppler methods had good accuracy in evaluating blood flow when suitably designed equipment was used in appropriate situations, with less than 6% systematic errors. However, measurement of cross-sectional area and beam angle correction led to random errors [33]. Fetal FO blood flow has been studied by Doppler ultrasound by some scholars [1, 13, 14], while these studies were mainly to establish the concept of fetal combined cardiac output, the research on FO blood flow was focused on the change trend of its proportion in combined cardiac output with gestational weeks, so that there was no specific value of FO blood flow for clinical reference. Therefore, the results of our study cannot be compared with these studies. Previously, FO blood flow was estimated indirectly by subtracting QP from LVCO. QP was expressed as the difference between the right cardiac output and the ductus arteriosus or the left and right pulmonary arteries. In these methods, the inner diameter and blood flow spectrum of multiple vessels need to be measured, there is no doubt that errors were inevitable. It was reported that errors in diameter measurement constitute the main obstacle to reliable flow measurement in small vessels when ultrasonographic techniques are used [39]. The inherent inaccuracy of these methods limits routine clinical practice. Our study shows that the direct measurement of blood flow in the FO is feasible. In our study, the four-chamber section which is used as the measured section is easily accessible. All the paraments needed to calculate FO blood flow volume can be obtained in a single section. The measurement errors resulting from multiple vessels inner diameters and beam angle adjustment in different sections are likely to be greatly reduced. Furthermore, the error caused by measurement in different periods in which the physiological state of the fetus is different can be reduced as much as possible. The QFO measurements obtained in this study showed good inter-observer reliability. Therefore, we believe that our current results provide an accurate presentation of QFO from 20 to 40 weeks of pregnancy and that the range provides a reasonable reflection of the biological variation. However, there are several limitations to the present work. The major limitation of the current study is its possible selection bias. FO-VTI was measured specifically in fetuses with only right-to-left shunting, contributing to the overestimation of QFO to some extent. The novelty of this approach warrants the reporting of this initial sample, and further studies with larger numbers of subjects will ensure the accuracy and reliability of the current study. Our initial experience suggests that this technique is best suited to fetus in the middle of gestation because adequate image acquisition and display with STIC are limited by early gestational age, fetal positioning, maternal obesity, and previous lower abdominal surgery, especially in the third trimester, when only 69% of data in the current were accepted despite the overall qualification rate of 80%, however, these disadvantages are also inherent to conventional ultrasonography. Fetal movement and sudden changes in FHR during data collection are additional factors affecting the technique, possibly causing serious mismatch of the information demanded for accurate reconstruction of moving cardiac structures.

## Conclusions

In summary, this study provides reference data on FO blood volume by means of pulsed Doppler combined with STIC. The findings of this study suggest that QFO increases with advancing gestational age, while iQFO decreases with advanced gestational weeks. The FO might have a limited capacity to increase its volume of blood flow during the last trimester. We believe that establishing the normal reference range of FO blood volume can not only improve the understanding of fetal circulatory physiology but also help to assess the risk level of the fetus at an early stage and facilitate perinatal management.

## Data Availability

The data and material in the current study are available from the corresponding author on reasonable request.

## Abbreviations

FO: Foramen ovale
LVCO: Left ventricular cardiac output
VTI: Velocity-time integral
QP: Pulmonary blood flow
4DUS: Four-dimensional ultrasound
STIC: Spatiotemporal image correlation
FHR: Fetal heart rate
2DUS: Two-dimensional ultrasound
FO-A: Foramen ovale area
FO-VTI: Foramen ovale flow velocity-time integral
EFW: Estimated fetal weight
ROI: Region of interest
QFO: Absolute blood flow of foramen ovale
iQFO: Weight-indexed foramen ovale blood volume
PC-MRI: Phase-contrast magnetic resonance imaging
